# Does prostate acinar adenocarcinoma with Gleason Score 3 + 3 = 6 have the potential to metastasize?

**DOI:** 10.1186/s13000-014-0190-z

**Published:** 2014-10-18

**Authors:** Rodolfo Montironi, Marina Scarpelli, Roberta Mazzucchelli, Antonio Lopez-Beltran, Matteo Santoni, Alberto Briganti, Francesco Montorsi, Liang Cheng

**Affiliations:** Section of Pathological Anatomy, Polytechnic University of the Marche Region, School of Medicine, United Hospitals, Via Conca 71, I − 60126 Torrette, Ancona, Italy; Unit of Anatomic Pathology, Department of Surgery, Faculty of Medicine, Cordoba, Spain; Fundação Champalimaud, Lisbon, Portugal; Medical Oncology, Polytechnic University of the Marche Region, School of Medicine, United Hospitals, Ancona, Italy; Department of Urology, IRCCS San Raffaele Hospital, Milan, Italy; Vita-Salute San Raffaele University, Milan, Italy; Department of Pathology and Laboratory Medicine, Indiana University School of Medicine, Indianapolis, IN USA

**Keywords:** Prostate cancer, Gleason score 6 adenocarcinoma, Gleason score, Lymphovascular invasion, Lymph node metastasis, Periprostatic lymph node

## Abstract

**Background:**

There is a worldwide debate involving clinicians, uropathologists as well as patients and their families on whether Gleason score 6 adenocarcinoma should be labelled as cancer.

**Case description:**

We report a case of man diagnosed with biopsy Gleason score 6 acinar adenocarcinoma and classified as low risk (based on a PSA of 5 ng/mL and stage cT2a) whose radical prostatectomy specimen initially showed organ confined Gleason score 3 + 3 = 6, WHO nuclear grade 3, acinar adenocarcinoma with lymphovascular invasion and secondary deposit in a periprostatic lymph node. When deeper sections were cut to the point that almost all the slice present in the paraffin block was sectioned, a small tumor area (<5% of the whole tumor) of Gleason pattern 4 (poorly formed glands) was found in an extraprostatic position.

**Conclusion:**

The epilogue was that the additional finding changed the final Gleason score to 3 + 3 = 6 with tertiary pattern 4 and the stage to pT3a.

**Virtual Slides:**

The virtual slide(s) for this article can be found here: http://www.diagnosticpathology.diagnomx.eu/vs/13000_2014_190

## Background

There is a worldwide debate involving clinicians, uropathologists as well as patients and their families on whether Gleason score 6 adenocarcinoma should be labelled as cancer. There are those in favor of continuing to call it cancer, while others are in favor of removing the label of cancer from Gleason 6 tumors [1-5]. Such hot debate is based on both clinical data and personal view.

Those who speak in favor of leaving the label of cancer base their opinion on the fact that Gleason score 6 cancer is composed of Gleason pattern 3 cancer, which shares cytological and molecular alterations associated with higher Gleason patterns and has the ability to extend locally beyond the prostate and metastasize [2]. Those who speak in favor of removing such label base their orientation on data demonstrating that using a time horizon of 10 to 15 years, less than 3% of men diagnosed with Gleason score 6 and classified as low risk will die as a result of prostate cancer whether treated or not [6]. A recent paper by Ross *et al*. showed that all adenocarcinomas of the prostate with Gleason Score ≤6 with lymph node metastasis had a higher grade component on slide review [7]. Finley *et al*. reported that one lymph node metastasis within the periprostatic fat was detected in a patient who had been preoperatively classified as low risk [8].

We report a case of man diagnosed with low risk Gleason score 6 acinar adenocarcinoma whose radical prostatectomy specimen was reported to harbor Gleason score 6 adenocarcinoma with lymphovascular invasion and metastasis in a periprostatic lymph node. The case presentation includes an epilogue that could better explain the tumor behavior.

## Case presentation

The patient, 67 years old, had a past 5-year history of increasing urinary obstructive symptoms and clinical diagnosis of benign prostatic enlargement for which he had been treated with an alpha(1A)/alpha(1D)-adrenoceptor antagonist. There was no history of other medications or hormonally-active supplements. No comorbidities, including diabetes, were present.

His most recent preoperative total serum PSA was 7.2 ng/ml, with a free-to-total ratio of 0.12. Transrectal ultrasound revealed a hypoechoic area in the left mid part of the prostate. Induration of the same prostate zone was detected by digital rectal examination. Twelve random ultrasound guided prostatic needle cores were taken. Histological diagnosis of Gleason score 3 + 3 = 6 acinar adenocarcinoma occupying 40% of one of the 12 cores, from the left part of the prostate and corresponding to the location of the hypoechoic area, was made. The tumor was classified in the low risk category. Even though the patient was considered a potential candidate for active surveillance, open radical prostatectomy (monolateral nerve sparing in the right side) was performed. Pelvic lymph node dissection was not carried out.

The prostate specimen was received fresh from the operating room. Its weight without the seminal vesicles (33 grams) and all three dimensions were recorded, the latter used for prostate volume calculation (24.12 cc). The specimen was then covered with India ink and fixed for 48 hours in 4% neutral buffered formalin. After fixation, the apex and base (3 mm thick slices) were removed from each specimen and examined by the cone method. The prostate body was step-sectioned at 3 mm intervals perpendicular to the long axis of the gland. The seminal vesicles were cut into two halves and processed *in toto*. The cut specimen was post-fixed and then dehydrated in graded alcohols, cleared in xylene, embedded in paraffin and examined histologically as 5 μm-thick whole-mount hematoxylin and eosin (H&E) stained sections. The Gleason grading was based on the 2010 modification of the “*2005 ISUP Modified Gleason System*” [9]. Staging was done according to the 2009 TNM revision [10].

**Figure 1:**
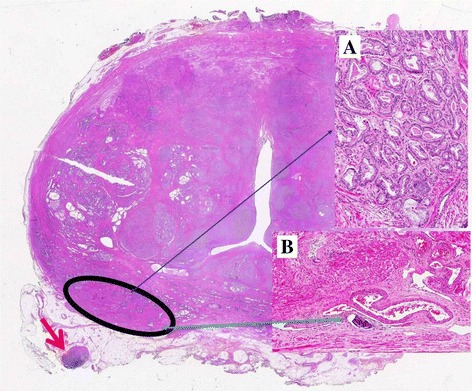
**Whole mount section.** The dominant nodule, contained in the circled area, with acinar adenocarcinoma Gleason score 6 (Insert **A**; thin bleu arrow) and lymphovascular invasion (insert **B**; thin green arrow). The adjacent periprostatic fat tissue shows a lymph node (thick red arrow) with metastasis (light pink part of the lymph node).

The histological examination of the prostate showed a dominant nodule located in the peripheral zone, postero-lateraly in the left mid portion of the prostate body (Figure 1). The nodule, 1.3 cm in greatest diameter (volume 0.7 cc), showed the following features: acinar adenocarcinoma (Figure 1A), Gleason score 3 + 3 = 6, WHO nuclear grade 3, negative surgical margin, no extraprostatic extension, even though the tumor reached the so-called prostate capsule, and lymphovascular invasion in the parenchyma adjacent to the tumor (Figure 1B). The left posterolateral periprostatic adipose tissue, adjacent to the prostate dominant cancer nodule, contained a lymph node, 0.5 cm in greatest diameter, with a secondary deposit of Gleason 6 adenocarcinoma (diameter 0.3 cm) (Figure 1, red arrow). An additional tumor focus was present in the right lateral horn with the features of an insignificant: Gleason score 3 + 3 = 6 acinar adenocarcinoma, 3 mm in diameter (volume 0.1 cc), with negative surgical margin and no extraprostatic extension (Stage pT2c) (See the Conclusions Paragraph).

## Discussion

The tumor was initially characterized by the following main features: Gleason score 3 + 3 = 6, WHO grade 3, lymphovascular invasion, and a lymph node with metastasis in the periprostatic soft tissue.

In our current presentation, the index tumor consisted of variably sized individual well-formed gland, without cribriform glands, individual cells and glomeruloid features, i.e., Gleason score 3 + 3 = 6. The same score was observed in the biopsy, dominant nodule of the specimen and lymph node deposit. The Gleason grading system of prostatic carcinoma is the quintessential prognostic factor in predicting findings in radical prostatectomy, biochemical failure, local recurrences, lymph node or distant metastasis in patients receiving no treatment, radiation therapy, radical prostatectomy and other therapies, including cryotherapy and high intensity focal ultrasound therapy [9,11-13]. Clinicians use routinely various tools, such as Partin tables or Kattan nomograms, to predict outcomes, including pathological stage or prognosis following treatment [14,15]. All of these tools incorporate the Gleason score.

The *International Society of Urological Pathology* convened a conference in 2005 in San Antonio, TX, USA, in an attempt to achieve consensus in controversial areas relating to the Gleason system. This has led to what is called “*2005 ISUP Modified Gleason System*” [12]. Lesions previously referred to as Gleason scores 2 to 4 in the classic system are now assigned a higher grade (Gleason score 6) in the modified system; however, those previously graded as Gleason score 6 in the classic system are often graded as Gleason score 7 tumors in the modified system. It has recently been recommended by Dr JI Epstein that all cribriform patterns are diagnosed as Gleason pattern 4 rather than pattern 3. It has also been suggests that glomerulations most likely represent an early stage of cribriform pattern 4 cancer and should likely be graded as pattern 4. Such recent changes are also known as 2010 modification of the “*2005 ISUP Modified Gleason System*” [9]. The general theme of such changes was to limit the definition of pattern 3 carcinoma and widen the scope of pattern 4 carcinoma.

**Figure 2:**
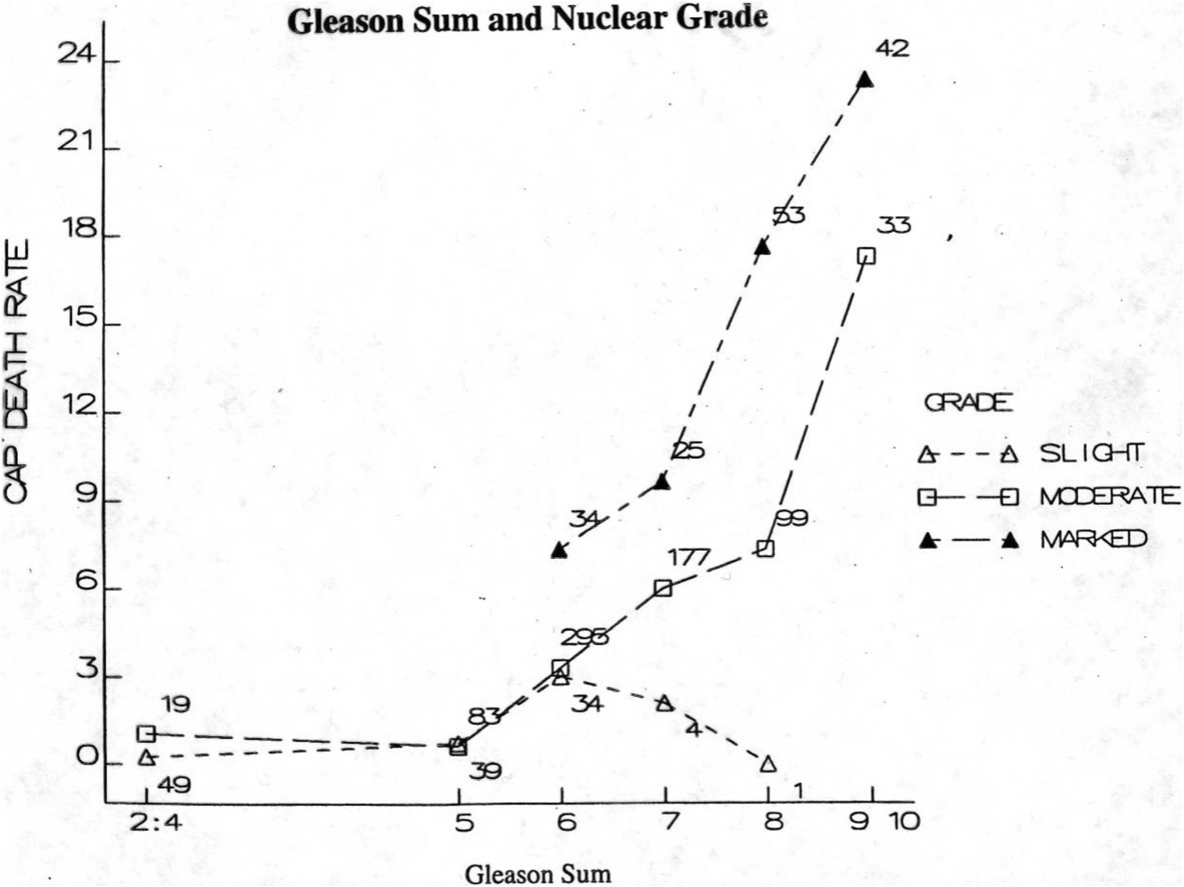
**This is a reproduction of the original diagram given by Dr Mostofi to one of the authors (RM).** Patients with Gleason score (i.e., Gleason sum) 6 and WHO high or marked nuclear grade have higher cancer of the prostate (CAP) death rate than those with lower grades (slight and moderate).

The Gleason system and its revisions are based on the architectural patterns of PCa. The contribution of nuclear morphology to its further refinement has been investigated only to a limited extent. It is worth mentioning the proposal made by Mostofi in 1999 in a WHO-sponsored meeting in Paris, France, to supplement the Gleason system with the WHO nuclear grading scheme [16]. The proposal was based on his and his group’s (in particular, Dr Isabel Sesterhenn’s) experience that patients with a Gleason score of 6 or higher cancers can be stratified based on the WHO nuclear grading and that such stratification has prognostic importance. As shown in the Figure 2, patients with Gleason score 6 and WHO nuclear grade 3 had significant higher cancer specific mortality than those with lower grades. This was recently confined in a morphometric study in which it was shown that the nuclear signature is important to better define risk groups in PCa patients [17]. In our current case the WHO grade was 3. Based on the findings of the present case, it is suggested that, in those patients who meet the current criteria for active surveillance, the tumor should also be evaluated for the presence of a WHO grade. If this finding is observed, the patient should be further evaluated for the presence of additional prognostic factors that can better define the aggressiveness of the lesion and require immediate therapy.

Our case showed LVI in the peritumoral parenchyma. LVI has been defined as the unequivocal presence of tumor cells within endothelial-lined spaces with no underlying muscular walls or as the presence of tumor emboli in small intraprostatic vessels. Baydar et al. [18] classified lymphovascular invasion as intratumoral (55%), at the periphery of the tumor (18%), both intra- and peritumoral (9%), and at a distant site from the tumor (18%). Univariate analyses showed lymphovascular invasion to be a significant predictor of disease recurrence and/or progression following radical prostatectomy and multivariate analyses have confirmed that lymphovascular invasion is an independent predictor of disease recurrence, when controlling for other pathologic variables known to influence clinical outcome. Studies have demonstrated an association between the presence of lymphovascular invasion and decreased time to biochemical progression, distant metastases, and overall survival after radical prostatectomy [18-20].

Our case showed metastasis in a periprostatic lymph node, i.e., in the periprostatic fat and in areas outside the standard lymphadenectomy. There are very few studies on the incidence and location of periprostatic/periseminal vesicle (PP/PSV) lymph nodes and the frequency of their involvement by metastatic PCa. In the study by Kothari et al. [21] 4.4% of patients showed PP/PSV lymph nodes. Sizes ranged from 0.7 to 4.5 mm (mean 1.8 mm). Distribution was 2 of 39 (5.1%) apical, 3 of 39 (7.7%) mid, 17 of 39 (43.6%) base, and 17 of 39 (43.6%) seminal vesicle. 0.6% had metastatic PCa to the PP/PSV lymph nodes. A recent study by Hansen et al. [22] found lymph nodes within periprostatic fat pads were detected in 5.5% patients, i.e., 19 patients. Metastasis was found in 4, only one with pT2a stage with Gleason score 3 + 4 = 7, the remaining 3 being pT3. Finley et al. reported that one lymph node metastasis within the periprostatic fat was detected in a patient who had been preoperatively classified as low risk, similar to our case [8]. These findings implicate that patients who seem to have low-risk PCa may harbor PCa metastases within lymph nodes of the periprostatic fat even if they do not have positive pelvic lymph nodes. Von Bodman et al and Briganti et al reported that the number of positive lymph nodes affects prognosis in PCa patients with lymph node metastases and reported cut-offs of 1 and 2 positive lymph nodes associated with adverse disease-related outcomes, respectively [23,24]. In this context, one positive lymph node within the periprostatic fat pad may influence a patient’s prognosis [22] and should be reported as N1 according to the AJCC Cancer Staging Manual [21].

## Conclusions

**Figure 3:**
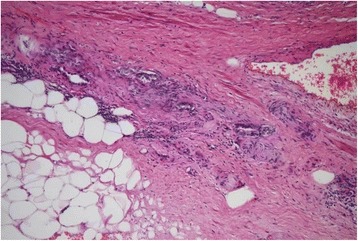
**Small tumor area (<5% of the whole tumor) of Gleason pattern 4 (poorly formed glands) in an extraprostatic position.**

When the paper based on the current case was considered ready for submission, we thought about a possible comment from a reader: Was the paraffin block containing the prostate slice with the dominant nodule and the lymph node serially sectioned to see whether the lesion changed in deeper sections? To give an answer to this possible question, deeper sections were cut to the point that almost all the slice was sectioned. We found a small tumor area (<5% of the whole tumor) of Gleason pattern 4 (poorly formed glands) in an extraprostatic position (Figure 3). This changed our final Gleason score to 3 + 3 = 6 with tertiary pattern 4 and the stage from pT2c to pT3a. This lead us to acquire some information on the follow-up of the patient whose radical prostatectomy was done in 2005. He is under complete androgen blockade therapy for a postoperative rising PSA levels and pelvic lymph node metastasis.

Our morphologic findings of Gleason score to 3 + 3 = 6 with tertiary pattern 4 with lymph node metastasis are in agreement with the histological observation made by Ross et al. [7]. They reviewed the slides of 17 radical prostatectomy specimens with Gleason Score (GS) ≤6 cancer and pelvic lymph node metastases. They found that 2 of their cases had tertiary pattern 4 with small cribriform glands. However, it was unknown whether it was the tertiary pattern 4 to confer the metastatic potential to the disease. An answer to this was given by Haffner et al. [25] in a recent study in which they used whole-genome sequencing and molecular pathological analyses to characterize the lethal cell clone in a patient who died of prostate cancer. They tracked the evolution of the lethal cell clone from the primary cancer to metastases through samples collected during disease progression and at the time of death. Surprisingly, these analyses revealed that the lethal clone arose from a small Gleason pattern 3 cancer focus in the primary tumor, and not from the bulk, higher-grade primary cancer or from a lymph node metastasis resected at prostatectomy.

## Consent

Written informed consent was obtained from the patient for publication of this case report and any accompanying images. A copy of the written consent is available for review by the Editor-in-Chief of this journal.
